# The interoceptive origins of mental imagery: an evolutionary account

**DOI:** 10.3389/fpsyg.2026.1807114

**Published:** 2026-03-10

**Authors:** Juha Silvanto

**Affiliations:** 1 Center for Cognitive and Brain Sciences, University of Macau, Taipa, Macao SAR, China; 2 Department of Psychology, Faculty of Health Sciences, University of Macau, Taipa, Macao SAR, China

**Keywords:** autonomic arousal, behavioral decision-making, forward models, interoceptive processing, motor imagery, social simulation, visual imagery

## Introduction

Contemporary theories suggest that mental imagery evolved to enable prospective simulation of future events for planning and decision-making (Suddendorf and Corballis, 2007; Schacter et al., 2008). Imagery allows organisms to construct hypothetical scenarios, enabling them to test alternative courses of action before acting (Moulton and Kosslyn, 2009). But how did mental imagery emerge from an evolutionary perspective?

I propose that imagery's origins lie in interoceptive processing, the phylogenetically ancient system linking internal bodily states to valence, emotion, and motivational salience (Craig, 2009; Barrett, 2017). Interoception is the necessary foundation for any planning system (such as imagery) because planning requires knowing what to plan for; which bodily and emotional states to pursue or avoid. Motor imagery emerged first, by extending forward models offline while inheriting interoceptive integration from survival-relevant actions. However, as social complexity in particular increased, organisms needed to simulate scenarios involving others (e.g., rival alliances, dominance conflicts, mating competitors) and here interoceptive signals alone proved insufficient, since the same autonomic arousal pattern can demand entirely different behavioral responses depending on context. Visual imagery evolved to solve this discriminability problem by binding distinctive sensory features with interoceptive states, creating multimodal representations where affective significance is constitutive of the image itself rather than a subsequent response to neutral sensory content.

## Evolutionary origins of imagery

Interoceptive processing emerged early in animal evolution (Ceunen et al., 2016) due to its foundational role in linking internal bodily states to valence and motivational importance. Nutritional resources are valued through hunger signaling, threats through autonomic arousal, reproductive opportunities through sexual arousal. With the evolution of more complex nervous systems, interoceptive processing came to constitute the foundation of emotions, emerging when interoceptive signals are integrated with contextual appraisal (Craig, 2009; Barrett, 2017). Because organisms act on what is survival-relevant rather than neutral information, any system evolved for prospective planning must be grounded in these interoceptive signals.

Motor simulation (the capacity to imagine actions before performing them for offline motor planning; Jeannerod, 2001) arguably represented the first form of imagery to emerge for two reasons. Firstly, motor actions most directly determine survival outcomes; selecting between fight or flight, pursuit or withdrawal, approach or avoidance carries immediate consequences. Secondly, motor imagery could build upon forward models already under selection for online motor control, the predictive systems that estimate sensory consequences of motor commands (Wolpert and Flanagan, 2001). Importantly, survival-relevant motor actions inherently involve interoceptive states such as cardiovascular arousal, respiratory load, autonomic preparation for fight, flight, or pursuit. Forward models for survival-relevant actions must predict both proprioceptive components (movement trajectories, body boundaries as points of contact) and interoceptive components (effort, threat, and pleasure) together; the former specifies what the body does, the latter specifies why it matters and whether the resulting state is worth pursuing or avoiding. When organisms evolved the capacity to generate these predictions offline for action selection, interoceptive components would be carried forward as part of the representational content being modeled. For example, when evaluating escape routes from a predator, accurate simulation requires predicting not only proprioceptive feedback (muscle tension, movement trajectory) but also interoceptive consequences (cardiovascular demands, respiratory load, sense of fear). Because survival-relevant motor actions already carried emotional significance and required autonomic preparation, motor imagery was scaffolded upon pre-existing interoceptive infrastructure. Evidence supports this architecture: motor imagery activates both motor cortex and autonomic systems, including the insula, the primary interoceptive hub (Tinaz et al., 2018; Jeannerod, 2001).

## From motor imagery to mental imagery

Motor simulation addressed challenges requiring coordinated action from a first-person perspective generally directly coupled to immediate motor execution. However, as social species faced increasingly complex pressures from group living, they needed to simulate social scenarios such as tracking rival alliances, predicting dominance conflicts, monitoring mating competitors, and coordinating group responses to threats. These scenarios carried significant survival consequences but required simulating events decoupled from immediate motor execution and involving spatial and social relationships beyond the agent's immediate action space. This decoupling does not mean that imagery lost its connection to action or bodily states. Rather, it follows naturally from the forward model architecture inherited from motor simulation, in which predictions can be generated and evaluated offline without triggering efferent consequences. Interoceptive responses to simulated scenarios became the mechanism for flexible behavioral decision-making.

Moreover, beyond discrete simulations, adaptive planning involves temporally-extended behavioral sequences. Interoceptive-sensory binding operates across these temporal structures. Planning a confrontation requires simulating the entire interaction sequence with interoceptive evaluation determining scenario interpretation (threat vs. opportunity), behavioral style (aggressive vs. appeasement), and affective coherence throughout implementation. These simulations are socially directed in that they are oriented toward modeling another agent's likely responses across the sequence, which is why interoceptive grounding is necessary. Furthermore, the simulation must track not just what happens but what it means for the individual's social standing or behavioral options.

However, the increasing complexity of simulation leads to what can be referred to as the discriminability problem. Autonomic states are low-dimensional in that cardiac acceleration, peripheral vasoconstriction, and cortisol elevation can occur in both encounters with dominants and competitive rivals, yet these scenarios demand different behavioral responses (submission vs. aggression) (Barrett, 2017). Thus interoceptive signals alone cannot specify which response is appropriate because the same bodily arousal pattern recurs across situations requiring different actions.

Visual features solve this problem by providing discriminative specificity. During actual social encounters, organisms learn associations between visual patterns (facial expressions, body postures, and individual identity), autonomic states, behavioral responses, and outcomes. For example, a dominant male's visual features become linked with autonomic arousal and successful submission; a rival's different features become linked with similar arousal but successful aggression. These experiences create bound memory traces where sensory, interoceptive, and behavioral components are stored as integrated units. During offline simulation, retrieving “the dominant male” activates a unified trace containing his visual features, the arousal pattern, and the submission response together as a single integrated representation. Empirical evidence supports this binding architecture. When organisms encounter novel stimuli, autonomic responses become integrated with sensory memory representations during encoding (e.g., Sokolov, 1963). Mental imagery reactivates these bound traces: imagining fearful stimuli triggers the same autonomic arousal and fear-related neural activity as actual perception, with stronger autonomic responses accompanying more vivid imagery (Agren and Hoppe, 2024). This demonstrates that accessing visual features during simulation typically activates their associated autonomic patterns because they were encoded together as integrated units.

But why did visual imagery need to retain this interoceptive integration from motor simulation? Without interoceptive signals, visual simulation would produce detailed but affectively neutral representations incapable of biasing behavior, providing no guidance on whether to approach or avoid. This functional requirement explains why visual imagery extended motor simulation architecture rather than evolving independently. Motor simulation had already solved the hard computational problems of generating predictions offline, triggering interoceptive/emotional states during simulation, binding sensory and interoceptive components into unified representations, and linking these to motor preparation systems. Visual imagery co-opted these existing solutions because both domains required transforming neutral sensory information into affective simulations that guide action selection.

## Functional and clinical implications

Why was embodied imagery adaptive? The key functional advantage is that it enables organisms to learn from imagined scenarios as if from direct experience. Because sensory and interoceptive components are bound during imagery construction, mental simulation generates the same bodily preparation states as actual encounters, allowing organisms to acquire threat contexts, approach opportunities, and behavioral strategies through imagination alone, without the costs and risks of trial-and-error learning. Consider a primate imagining rivals at territorial boundaries. Sensory-interoceptive binding in mental imagery creates a unified representation integrating location cues, threat assessment, and autonomic arousal. When the animal later approaches an actual boundary, environmental cues automatically trigger heightened vigilance through pattern completion, even without rivals present. The individual has learned threat contexts through imagination, enabling adaptive preparation without direct experience. This learning mechanism is not limited to direct experience: observing conspecifics in threat or reward contexts generates autonomic responses in the observer, creating bound sensory-interoceptive traces through vicarious experience. Social learning, narrative, and fictional representation thus feed into the same binding architecture, allowing individuals to acquire affectively-grounded simulations from indirect sources.

One might argue that as social learning becomes more important, interoceptive processing becomes less central since behaviors become rule-governed and automatic. However, well-learned responses become automatic only in familiar contexts. Imagery remains relevant when situations are novel, ambiguous, or involve conflicting learned rules that require simulation to evaluate appropriate responses.

This bound nature of imagery (cf. Silvanto and Nagai, 2025; Scholz et al., 2026) contrasts with sequential models such as Lang (1977, 1979) bio-informational theory, where imagery is purely sensory and autonomic responses follow as separate consequences. Sequential processing creates two vulnerabilities: partial environmental cues may fail to activate the complete sensory representation needed to initiate the causal chain, and even when successful, additional processing steps introduce delays that matter in survival contexts where rapid behavioral responses determine outcomes. Bound representations instead enable environmental cues to directly trigger integrated preparation states through pattern completion, a distinction with testable empirical consequences. Recent empirical evidence is consistent with the latter view. If interoceptive processing is integral to imagery construction rather than a subsequent consequence, then disrupting interoceptive systems should directly impair imagery generation. Consistent with this, conditions involving autonomic dysfunction (hypermobility, postural orthostatic tachycardia syndrome) relate to imagery capacity (Nagai et al., 2025). These are physiological conditions which cannot reasonably be attributed to reduced mental imagery, suggesting a causal influence from autonomic function to imagery rather than the reverse. Similarly, childhood trauma reduces imagery vividness across sensory modalities (Gao et al., 2025), with evidence that this effect is mediated by trauma-induced autonomic dysregulation.

The link between psychological trauma and loss of imagery exemplifies how sensory-interoceptive binding creates vulnerability when bodily signals and social requirements chronically diverge. Imagery evolved assuming alignment between bodily signals and adaptive responses (e.g., threat-arousal leading to escape, attraction to approach). However, social environments can create chronic misalignment. When threat-arousal must be repeatedly suppressed (e.g., abuse demanding submission rather than escape), interoceptive signals predict behaviors the social environment prohibits, creating sustained predictive conflict. In this case, imagery generates chronically distressing simulations (e.g., vivid embodied representations of escape or resistance that cannot be enacted). Under these conditions, downregulating imagery's interoceptive components may be adaptive, leading to a more abstract form of mental representations.

This architecture specifically evolved to generate experiential mental imagery (vivid sensory simulations with phenomenological character) rather than abstract spatial cognition. Mental rotation, navigation planning, and symbol manipulation represent distinct computational systems that process spatial relationships without phenomenological vividness, as evidenced by aphantasic individuals who retain these abilities while completely lacking imagery (Zeman, 2024). This dissociation supports the framework: experiential imagery requires interoceptive grounding because its adaptive function was evaluating emotionally significant scenarios. However, interoceptive grounding likely varies continuously across representational contexts, with abstract spatial cognition representing the lower end of this continuum rather than a categorically separate process.

## Conclusion

While grounded cognition has established that imagery draws on perceptual, motor, and interoceptive systems (see e.g., Barsalou, 2008; Muraki et al., 2023; Silvanto and Nagai, 2025), the evolutionary sequence through which imagery emerged or why it should integrate these particular modalities has remained unaddressed. I propose that interoception served as the evolutionary foundation from which motor and then visual imagery developed. Motor imagery emerged by extending forward models offline while inheriting interoceptive integration from survival-relevant actions. Visual imagery extended this architecture because both domains faced the same functional challenge of transforming neutral sensory information into affectively weighted simulations that guide action selection. This functional overlap resulted in sensory and interoceptive components being bound during imagery construction, creating multimodal representations where mental images inherently possess affective significance.
